# Cognitive impairment profile in adult patients with Niemann pick type C disease

**DOI:** 10.1186/s13023-017-0714-1

**Published:** 2017-10-18

**Authors:** Camille Heitz, Stéphane Epelbaum, Yann Nadjar

**Affiliations:** 1 Neurology Department, Hôpital Universitaire de Nîmes, Nîmes, France; 2 Neurology Department, Reference Center for Lysosomal Diseases, Hospital of Pitié-Salpêtrière, Paris, France

**Keywords:** Niemann pick type C disease, Lipidose, Neuropsychological assessment, Cognitive impairments

## Abstract

**Background:**

Cognitive impairment is one of the core symptoms of Niemann Pick type C (NPC) disease, but few data concerning the neuropsychological profile of NPC patients are available. The aim of our study was to characterize cognitive impairments in NPC disease and to assess the evolution of these symptoms and the impact of miglustat on cognitive follow-up.

**Methods:**

We conducted a retrospective study of 21 adult patients diagnosed with NPC disease. Neuropsychological data (global cognitive efficiency, language, attention, executive functions, praxis, and visuoconstructive functions tests) were retrieved to describe the cognitive profile of patients. Cognitive impairment scores over time were assessed under treatment by miglustat.

**Results:**

The majority of patients (90%) were impaired in one or more cognitive function. Executive functions and attention were the most impaired cognitive functions. Conversely, storage in the episodic memory was preserved in 61.5% of cases. Mean neuropsychological scores were stable during the period under miglustat (mean of 3.8 years).

**Conclusions:**

This study is one of the first to assess the cognitive profile of adult NPC patients. This profile is not specific to attention and executive dysfunctions; however, the preservation of storage in the episodic memory is promising for cognitive remediation. Further studies are needed to confirm the role of miglustat on cognition, but in this study, patients were stable under this treatment.

## Background

Niemann Pick type C (NPC) disease is a lipid storage disorder characterized by visceral (hepatosplenomegaly) and neurological symptoms (ataxia, dystonia, cognitive disorder, psychiatric disorder, and vertical supranuclear gaze palsy). It is an autosomal recessive disease caused by mutations in either of the two genes encoding the lysosome-associated lipid trafficking proteins, NPC1 and NPC2, with an occurrence estimated as 1/120,000 individuals in Western Europe [1]. The onset is typically in childhood, but NPC may also have late onset in adulthood. Neurologic and psychiatric symptoms are common in the adult form. Miglustat, a glucosylceramide synthase inhibitor, has been shown to alleviate some of the neurological symptoms [2].

Cognitive impairment is one of the core symptoms of this disease, with 86% of NPC patients (children and adults) presenting cognitive impairments [3], which appear early in the course of the disease. However, few data concerning the neuropsychological profile of NPC patients are available. To our knowledge, only one study on a small (10 patients) cross-sectional cohort has assessed cognitive impairments observed in adult NPC patients [4]. In addition, the impact of miglustat has never specifically been studied on cognitive disorders in adult NPC patients. Further evaluations of cognitive impairments due to NPC disease are important to understand patients’ difficulties, to adapt cognitive remediation or to organize help at school or work. Moreover, comprehensive data on cognitive disorders in adult NPC patients are needed to correctly monitor cognitive functions in therapeutic trials. For instance, the mini mental state evaluation (MMSE) [5], which explores global cognition, is probably not a sensitive tool to detect cognitive changes in NPC adult patients. A better description of cognitive profile would be useful for further therapeutic studies.

The aim of our study was to describe cognitive impairments in NPC adult patients and to assess their stability over time.

## Methods

We conducted a retrospective study of adult NPC patients followed in the Neurology Department of Hospital of Pitié-Salpêtrière (Paris, France), between 1997 and 2015. All included patients were all genetically confirmed in adulthood (>15 years old), all harbouring one homozygous or two heterozygous NPC1 mutations. We included patients who performed at least one neuropsychological assessment during their follow-up. A letter from the CRML (Centre de reference des Maladies Lysosomales; reference center for lysosomal diseases) was sent to each patient to inform them of the collection of data from their clinical charts, with a contact given for further information or to express refusal. The CPP (Comité de Protection des Personnes) in La Salpétrière Hospital (local ethic committee) approved this study.

The following data were retrieved from the patient’s medical files: gender, age, education level, disease duration, presence of a psychiatric disorder, clinical symptoms, neuropsychological assessment, and the date of miglustat initiation. Clinical scores were also retrieved at time of diagnosis, as defined in Iturriaga et al. [6]. Clinical score is a six-item scale concerning ambulation, manipulation, speech disorder, swallowing disorder, oculomotor disorder, and epilepsy. For most patients, clinical scores were retrospectively calculated on the basis of clinical charts.

The neuropsychological assessment comprised global cognitive scales (MMSE, Mattis dementia rating scale [7], executive functions (FAB (frontal assessment battery [8], Wisconsin card sorting test [9], lexical and semantic fluency), attention (verbal and visual span), memory (FCSRT (free and cued selective reminding test) [10, 11], language (BNT (Boston naming test) 34 items [12], and visuoconstructive functions (Rey-Osterrieth complex figure test [13] evaluations. Spans assess the working memory, whereas FCSRT assesses the three components of episodic memory: encoding, storage and retrieval.

Alterations in the severity of cognitive impairments were assessed under miglustat. We also evaluated correlations between neuropsychological scores and clinical data (age of onset, age of the first neuropsychological evaluation, disease duration, level of education, presence of psychosis, score of walking/manipulation/dysarthria/deglutition, time between the onset of disease and the initiation of miglustat, and duration of miglustat).

The Statistical Package for Social Sciences software (SPSS ver. 21.0, Wellcome Department of Imaging Neuroscience, London; http://www.fil.ion.ucl.ac.uk/spm) was used for statistical evaluation as required. Comparison of first and last neuropsychological exams was assessed using Wilcoxon test. Correlations between clinical data and neuropsychological tests were assessed by Pearson’s test. All cognitive variables are treated as continuous. For each test, a probability value of < 0.05 was considered significant.

All of the data were generated during a routine clinical work-up and were retrospectively extracted for the purpose of this study. Therefore, according to French legislation, explicit consent was waived. However, regulations concerning electronic filing were followed, and patients and their relatives were informed that anonymized data might be used in research investigations and particularly for the present study.

## Results

### Population characteristics

**Fig. 1:**
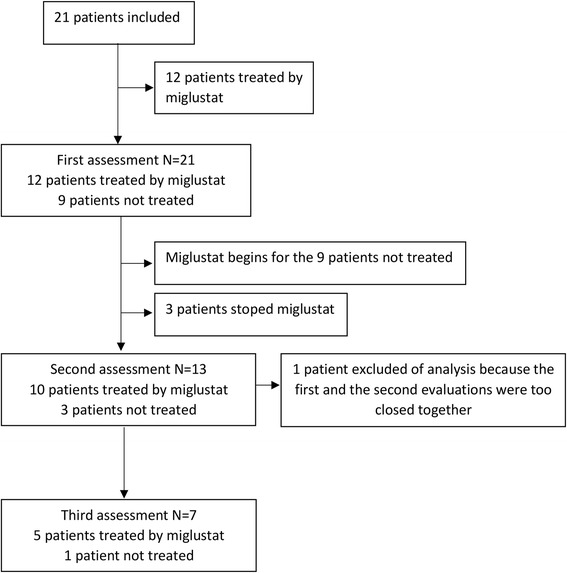
Study flowchart. This diagram represents the study design with the inclusion of patients and the three assessments

We studied 28 patients’ medical records and a total of 21 patients were included into the study (Fig. 1). The remaining seven patients were excluded because they did not undergo a neuropsychological evaluation.

Population characteristics are presented in Table 1. The mean age was 34 years old at first cognitive evaluation, with a female predominance (62%). Six patients discontinued their education in primary school and five patients completed high school. Some patients were treated by medications (four patients by antipsychotics, three patients by anticonvulsants, seven patients by benzodiazepines, four patients by antidepressants).

**Table 1:** Population characteristics at the first examination

	Mean	SD	Median	IQR
Age (years)	34.9	12.6	30	24.5–40.5
Gender (M/F)	8/13			
Education (years)	9	3.2	11	5–12
Age of onset (years)	21.6	15.4	16	13.3–25
Disease duration (years)	14.4	13.2	9	4–19
Walking score (/5)	1.4	0.8	1	1–2
Manipulation score (/4)	1.6	0.6	2	1–2
Dysarthria score (/5)	1.8	0.7	2	2–2
Deglutition score (/4)	1.5	1.1	2	0–2
History of psychosis Y/N	7/14			
Learning disorder Y/N	3/18			
Time between the onset of disease and the initiation of miglustat (years)	7.7	5	7	3.7–17.7
Duration of miglustat (months)^a^	18.8	15.5	18	3–32.3

A total of 21 patients were included in the study. Disease duration is the time between the onset of the disease and the first neuropsychological examination. Walking, manipulation, dysarthria and deglutition scores are determined at diagnosis. All patients were treated by miglustat but three patients stopped the treatment after 5, 6 and 13 months of treatment respectively

^a^Duration of therapy prior to first neuropsychological assessment

*IQR* interquartile range, *M/F* male/female, *SD* standard deviation, *W* women, *Y/N* yes/no.

Eight patients had one neuropsychological evaluation, six patients had two neuropsychological examination and seven patients had three neuropsychological examinations. For three patients, the first assessment was performed before the diagnosis of NPC (mean of 32.6 months). For other patients, the first assessment was performed an average of 23.9 months after the diagnosis. The second assessment was performed an average of 44 months after diagnosis and the third assessment an average of 80.6 months after diagnosis. In total, 57% of patients (*N* = 12) were treated by miglustat at the time of the first neuropsychological examination. All other patients (*N* = 9) initiated miglustat treatment after the first neuropsychological evaluation. Three patients stopped miglustat before the second neuropsychological examination because of side effects. In total, 10 patients were treated by miglustat at the time of the second neuropsychological test.

### Neuropsychological impairments in NPC

**Fig. 2:**
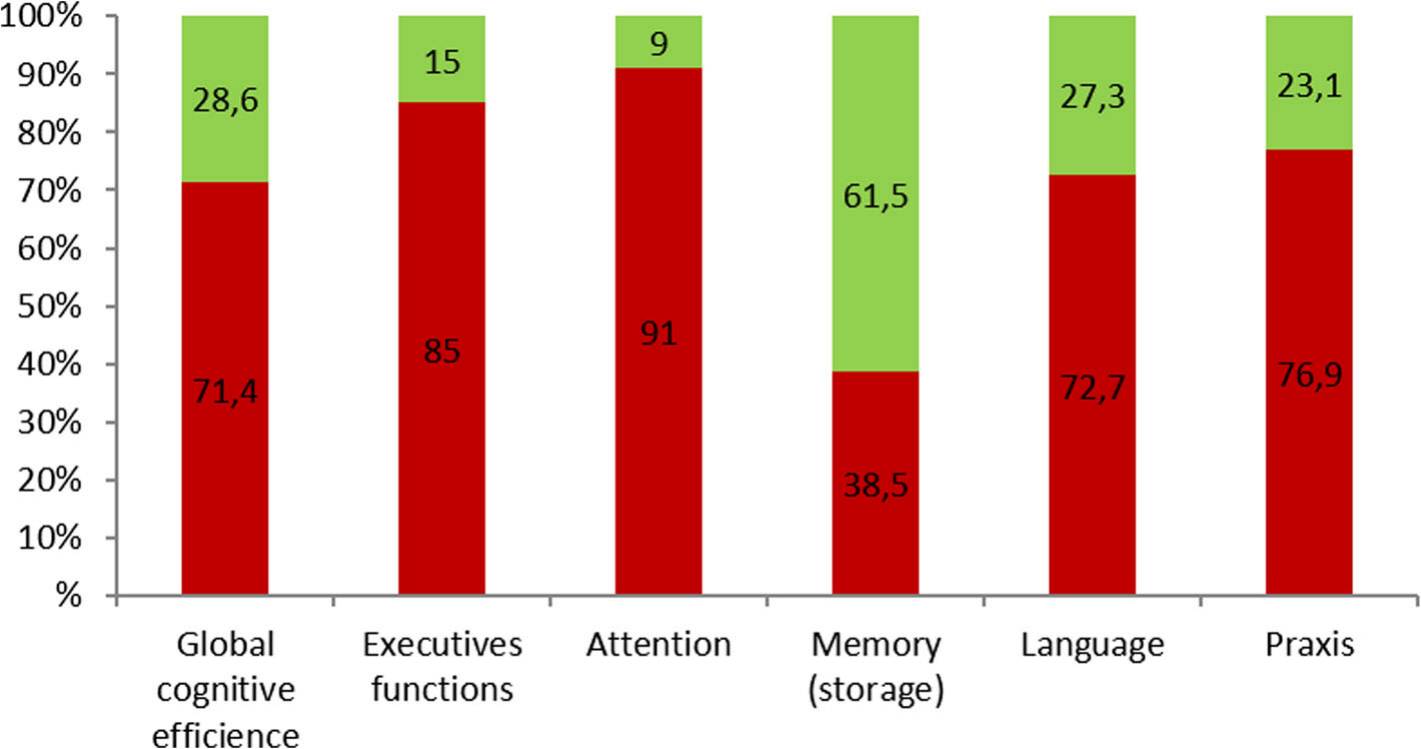
Impairments of the main cognitive functions. The figure represents the percentage of patients with impairment (red) or preservation (green) of each cognitive function for the first neuropsychological examination (*N* = 21 patients)

The majority of patients (90%) were impaired in one or more cognitive function (Fig. 2). Cognitive functions were preserved for only two patients. Impairments were more frequent for attention and executive functions. Global cognitive efficiency, language and praxis were impaired in three-quarters of patients. In contrast, storage in episodic memory was preserved in more than half of the patients.

**Fig. 3:**
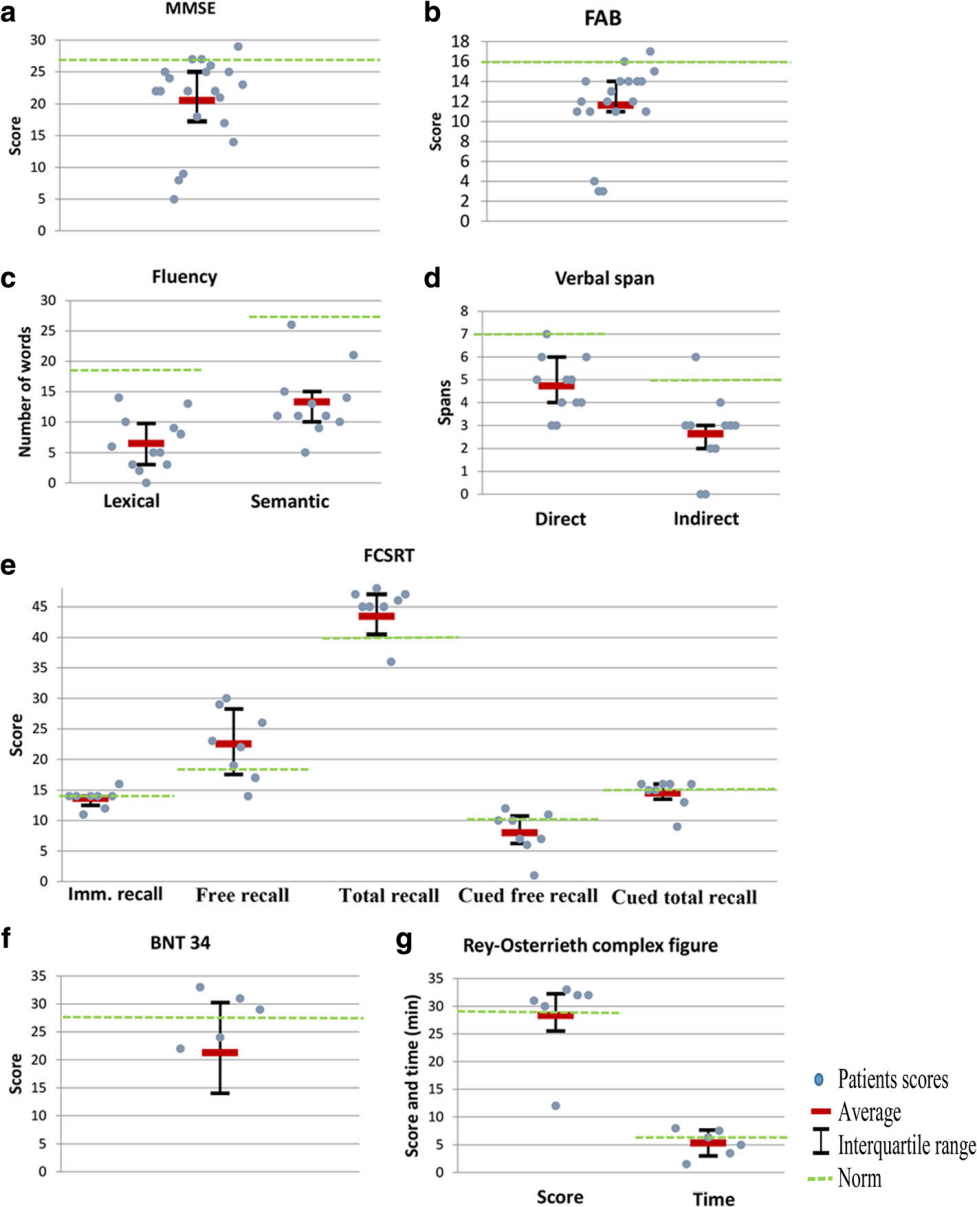
Mean and interquartile ranges of neuropsychological test scores. This figure presents patients’ scores, averages and interquartile ranges of tests on the first neuropsychological examination. BNT = Boston naming test; FAB = frontal assessment battery; FCSRT = free and cued selective reminding test; MMSE = mini-mental state examination

The results of neuropsychological tests are presented in Table 2 and Fig. 3. The duration of treatment with miglustat was on average 18.8 months (SD = 15.5) in the 12 patients who were already treated with miglustat at the first neuropsychological examination. The mean MMSE was 20.6 (SD = 6.8). Executive functions were impaired for 17 patients (85%), and the mean FAB was 11.6 (SD = 4.1). Attention was impaired for direct spans, with patients able to memorize 4.7 ± 1.3 words or 4.1 ± 1.5 pictures (normal range = 7 ± 2). Similarly, working memory deficits were observed for indirect spans, with patients able to memorize 2.6 ± 1.7 words or 3.2 ± 1.8 pictures (normal range = 5 ± 2). Concerning memory, patients were more impaired for spontaneous (mean free recall [FCSRT]: 22.5 ± 5.7) than for cued retrieval (mean total recall [FCSRT]: 43.4 ± 5.6), consistent with the executive function changes.

**Table 2:** Neuropsychological evaluation in NPC patients

Functions	Tests	n	Mean	SD	Median	IQR	Norms^a^
Global cognitive efficiency	Impaired (Y/N)	17/4					
	MMSE (/30)	20	20.6	6.8	22	17.2–25	≥ 27
	Mattis (/140)	12	103.6	28.6	117	110–125	≥ 123
Executive functions	Impaired (Y/N)	17/3					
	FAB (/18)	19	11.6	4.1	12	11–14	≥ 16
	Wisc. (criteria) (/6)	9	3.6	1.9	3	2–6	≥ 5.6
	Lexical fluency	12	6.5	4.4	5.5	3–9.7	≥ 17–19
	Semantic fluency	11	13.3	5.8	11	10–15	≥ 26–29
Attention	Impaired (Y/N)	11/1					
	Direct verbal spans	11	4.7	1.3	5	4–6	7 ± 2
	Indirect verbal spans	11	2.6	1.7	3	2–3	5 ± 2
	Direct visual spans	9	4.1	1.5	4	3–5	7 ± 2
	Indirect visual spans	9	3.2	1.8	3	2–4.5	5 ± 2
Memory (FCSRT)	Impaired (Y/N)	6/7					
	Immediate Recall (/16)	8	13.6	1.5	14	12.5–14	≥ 14
	Free recall (/48)	8	22.5	5.7	22.5	17.5–28.2	≥ 17
	Total recall (/48)	9	43.4	5.6	45	40.5–47	≥ 40
	Cued free recall (/16)	8	8	3.6	8.5	6.2–10.7	≥ 10
	Cued total recall (/16)	8	14.5	2.5	15.5	13.5–16	≥ 15
Language	Impaired (Y/N)	8/3					
	BNT 34 (/34)	4	21.3	8.7	19.5	14–30.2	≥ 27
Praxis	Impaired (Y/N)	10/3					
Visuo-contructive functions	Impaired (Y/N)	5/5					
	Rey figure (score)	6	28.3	8.1	31.5	25.5–32.2	≥ 29
	Rey figure (second)	6	319.2	149.9	341	179.5–459.7	≤ 360

This table presents results of the first neuropsychological examination. This test took place an average of 14.4 years after the onset of the disease and 16.6 months after the diagnosis

*BNT* Boston naming test, *FAB* frontal assessment battery, *FCSRT* free and cued selective reminding test, *IQR* interquartile range, *MMSE* mini-mental state examination, *N* number of available tests, *SD* standard deviation, *Y/*: yes/no.

^a^These are approximated norms for patients between 30 and 40 years old to *give an approximate cut-off of* expected results

We found significant correlations between the MMSE score and age (R^2^ = −0.57, *p* = 0.01) and disease duration (R^2^ = −0.49, *p* = 0.03) (Table 3). MMSE was also correlated with the dysarthria score (R^2^ = 0.49. *p* = 0.05) and manipulation score (R^2^ = 0.51. *p* = 0.04). The FAB score was also correlated with the dysarthria score (R^2^ = 0.59. *p* = 0.02). We also found a correlation between lexical fluency and presence of psychosis (R^2^ = 0.6. IC95 = 0.2–0.9). Praxis were correlated with disease duration (R^2^ = 0.7. IC95 = 0.5–0.9).

**Table 3:** Correlation between MMSE, FAB and clinical data

	MMSE	FAB
Age of onset	R^2^ = −0.04 (*p* = 0.87)	R^2^ = −0.3 (*p* = 0.23)
Age at first examination	**R** ^**2**^ **= −0.57 (** ***p*** ** = 0.01)**	R^2^ = 0.27 (*p* = 0.28)
Disease duration	**R** ^**2**^ = **−0.49 (** ***p*** **= 0.03)**	R^2^ = −0.07 (*p* = 0.77)
Education level	R^2^ = 0.10 (*p* = 0.69)	R^2^ = 0.14 (*p* = 0.59)
Presence of psychosis	R^2^ = 0.27 (*p* = 0.28)	R^2^ = 0.20 (*p* = 0.41)
Walking score	R^2^ = 0.2 (*p* = 0.45)	R^2^ = 0.19 (*p* = 0.48)
Manipulation score	**R** ^**2**^ ** = 0.51 (** ***p*** **= 0.04)**	R^2^ = 0.12 (*p* = 0.66)
Dysarthria score	**R** ^**2**^ **= 0.49 (** ***p*** **= 0.05)**	**R** ^**2**^ **= 0.59 (** ***p*** **= 0.02)**
Deglutition score	R^2^ = 0.2 (*p* = 0.45)	R^2^ = 0.13 (*p* = 0.65)
Duration of miglustat	R^2^ = −0.46 (*p* = 0.1)	R^2^ = −0.17 (*p* = 0.57)
Time between the onset of disease and the initiation of miglustat	R^2^ = −0.28 (*p* = 0.25)	R^2^ = 0.1 (*p* = 0.71)

A probability value of <0.05 was considered significant

Significant results are written in bold

*FAB* frontal assessment battery, *MMSE* mini-mental state examination

**Fig. 4:**
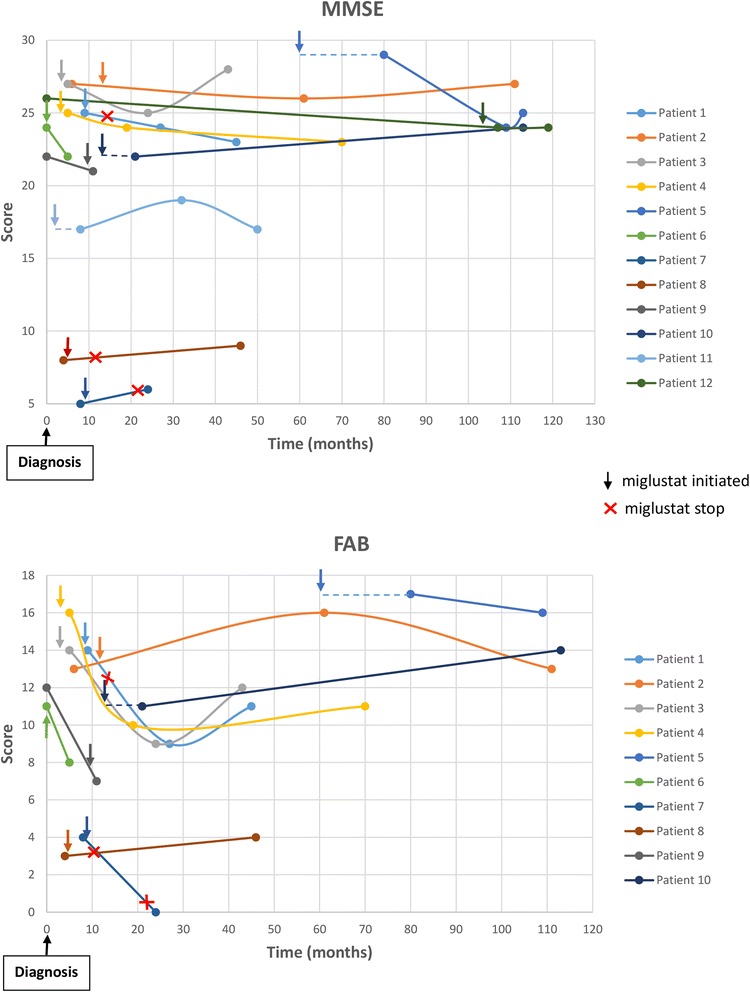
Evolution of FAB and MMSE with treatment by miglustat. This figure presents the MMSE and FAB scores over time with treatment by miglustat. The initiation of miglustat is indicated by an arrow. Each point represents a neuropsychological examination. Patient 8 had learning disabilities. Dotted lines denote patients who were treated by miglustat before their first neuropsychological evaluation. FAB = frontal assessment battery; MMSE = mini-mental state examination

In the 12 patients (for MMSE) and 10 patients (for FAB) who underwent two or more neuropsychological exams, we studied the neuropsychological exam scores over time (Fig. 4). Five patients (patients 2, 3, 4, 10 and 11) were treated by miglustat for more than 40 months and were stable (+/− 2 points on MMSE) [14], these patients had cognitive impairments at the baseline, with MMSE between 17 and 27. Conversely, patient 12 was stable (−2 points on MMSE) for 100 months but was treated by miglustat only at the end, and patient 5 lost 4 points on MMSE while treated by miglustat for 50 months.

Comparison of test scores between the first and the last neuropsychological examinations is shown in Table 4. We excluded the three patients who stopped miglustat between the first and the second neuropsychological examinations. The mean duration between the two tests was 56.4 months (SD = 41.3). Five patients were treated by miglustat at the first evaluation and all patients were treated by miglustat at the last evaluation. Results were worse at the last neuropsychological examination for global cognitive functions, executive functions and attention, but differences were not significant (*p* > 0.05). Memory functions were very stable between the two evaluations.

**Table 4:** Evolution of neuropsychological tests between the first and last evaluation

Functions	Tests	N	First test mean (SD)	Last test mean (SD)	*p*
Global cognitive efficiency	MMSE (/30)	10	24.4 (3.4)	23.5 (3.1)	0.168
	Mattis (/140)	6	119 (5.7)	113.5 (10.3)	0.115
Executive functions	FAB (/18)	8	13.5 (2.2)	11.9 (3.1)	0.140
	Wisconsin (errors)	4	4 (5)	5.5 (6.9)	0.465
	Wisconsin (criteria)	4	3.4 (2.4)	3.8 (2.7)	0.655
	Lexical fluency	6	7.7 (3.1)	5.3 (1.9)	0.104
	Semantic fluency	6	12.7 (4.4)	11 (3.7)	0.673
Attention	Direct verbal span	5	5 (0.7)	4.4 (0.5)	0.180
	Indirect verbal span	4	3 (0.8)	3.5 (0.6)	0.157
	Direct visual span	3	4.7 (0.6)	4.7 (0.6)	1
	Indirect visual span	3	4 (1)	4.3 (0.6)	0.317
Memory (FCSRT)	Immediate Recall (/16)	6	14 (1.3)	14.2 (1.2)	0.564
	Free recall (/48)	6	20.2 (4.4)	17.8 (6.7)	0.136
	Total recall (/48)	6	44.8 (4.4)	43.2 (4.4)	0.102
	Delayed free recall (/16)	6	7 (3.5)	7.8 (4.1)	0.595
	Delayed total recall (/16)	6	14.3 (2.9)	14.3 (4.1)	1

The mean duration between two tests was 56.4 months (SD = 41.3). One patient had learning disabilities. Five patients were treated by miglustat at the first evaluation and all patients were treated by miglustat at the last evaluation

*FAB* frontal assessment battery, *FCSRT* free and cued selective reminding test, *MMSE* mini-mental state examination, *N* number of available tests, *SD* standard deviation

## Discussion

The purpose of this study was to assess cognitive functions in adult NPC patients. Few studies have explored cognitive functions in this disease. This study, on 21 patients, represents a large cohort for such a rare disease. Moreover, 12 of those patients had more than one cognitive evaluation with a long follow-up (mean 5.2 years) and some of them had neuropsychological evaluation before and after miglustat treatment.

Results showed that executive functions and attention were the most impaired functions. Indeed, FAB, visual and verbal span were impaired in the majority of patients. These results are comparable with the description of NPC patients by Sévin et al. [15]. Indeed, 61% of their patients had cognitive impairments, with mainly executive dysfunctions and frontal syndrome. They also reported aphasia, apraxia and memory impairment for some patients. Several studies have showed frontal hypometabolism in NPC patients, likely responsible for the attention and executive dysfunctions [16–18]. Walterfang et al. [19] showed a reduction of gray matter in the thalamus, bilateral hippocampus, insula, striatum and cerebellum, as well as widespread reductions in fractional anisotropy in major white matter tracts. Atrophy in these regions was correlated with global cognitive score, particularly for the hippocampus and the thalamus [20]. Hippocampal and thalamic atrophy could explain cognitive impairments in NPC disease.

In our study, storage in the episodic memory was preserved in 61.5% of cases with an impaired free recall and a preserved total recall on the FCSRT. These results are interesting because NPC and Alzheimer’s disease (AD) histology share common neuropathological features. Indeed, studies have shown alterations of APP metabolism involving the intracellular accumulation of amyloid β (Aβ) in NPC [21, 22]. NPC brains also contain neurofibrillary tangles formed by hyperphosphorylated microtubular protein tau, similar to that in AD [22]. However, Purkinje cells in the cerebellum are the most affected neurons in NPC, while lesions are preferentially localized in medial temporal lobes in AD. NPC disease and AD share some pathophysiological similarity*,* but cognitive profiles are very different. Our hypothesis to explain this result is that cognitive symptoms are not only lesion-driven (Aß & Tau) but are also linked to the localization of cerebral damage and to the progression of brain lesions.

The neuropsychological profile in NPC is not specific to this disease; executive and attention dysfunctions are found in many neurological diseases. Cognitive impairment is related to disease duration and disease severity (particularly for MMSE and praxis). Klarner et al. [4] found equivalent results and proposed more suitable tests to assess NPC according to the stage of the disease. For example, the Trails Making Tests and Grooved Pegboard tests were best suited for patients in the earlier stages of the disease, whereas the Find Similarities test is more appropriate for later stages of the disease, when the patients’ severe lack of fine motor skills preclude the use of the first two tests.

The relative preservation of storage in the episodic memory is interesting for the cognitive remediation of patients. Rehabilitation procedures for attention and executive functions would be done with a good storage of these information and so probably with a good efficacy. Rehabilitation may be particularly useful in these patients, who mostly showed no deterioration of cognitive impairment over several years.

Klarner et al. [4] assessed cognitive functions in NPC. They found impairments in fine motor skills, language, attention, working memory, and visuospatial functions. Only MMSE and constructional praxis were preserved in 50% of cases. Cognitive impairments were related to the stage of the disease. However, their study was performed on a small number of patients (eight men and two women) and tests were not available for all patients. In another study, Klarner et al. [23] report 14 NPC patients (ages 12–43 years) with impairments principally in executive functions, which is concordant with our findings. Apart from these two works, no other studies have assessed neuropsychological profile in NPC. A recent publication [24] reviews the cognitive profile of 117 NPC adults patients (case reports). Among these patients, characterization of cognitive deficits was available for only 23 patients, with few data. Executive dysfunction was the most frequent feature which is in line with our study. Authors highlighted the probable role of cerebellum and deep gray matter nuclei (thalamus, striatum) in these features.

One of the main interests of our study was to track neuropsychological test scores under miglustat for 12 patients. We did not find any significant improvement of cognition after miglustat therapy. This might be due to the small number of patients and the retrospective nature of the study. Conversely, we did not find a significant decline in cognition after miglustat either, suggesting that patients remain globally stable under treatment for several years. Follow-up of MMSE and FAB scores showed stability of scores after the initiation of miglustat for the majority of patients. Wraith et al. [25] found similar results for MMSE score over time in a prospective study. In 18 patients who completed 12 months of miglustat therapy, mean MMSE scores were 22.94 at baseline and 24.06 at Month 12. Of the six patients with available data who completed 24 months of miglustat therapy, mean scores were 19.50 at baseline, 21.17 at Month 12, and 19.33 at Month 24. Patterson et al. also found a slight improvement of the mean MMSE after 12 months of miglustat [26]. Stability of cognitive features under miglustat is maybe due to the slower rate of white matter change in the corticospinal tracts, the thalamic radiation and the inferior longitudinal fasciculus in treated patients [27]. In the same way, Masingue et al. [28] studied evolution of atrophy and fractional anisotropy in NPC patients. They found significant atrophy in basal ganglia, cerebral peduncles and corpus callosum for NPC patients compared to controls. With miglusat, no volumetric change was observed.

Our study had some limitations. Principally, it was a retrospective study and neuropsychological examination was not fully standardized. In consequence, some data were not available, particularly for language and visuospatial functions. However, the relatively large number of participants, the length of follow-up and the fact that this study reflects the clinical routine (i.e. less selected patients than in clinical trials) give new insights into the benefits of miglustat and the possibility of cognitive rehabilitation in NPC patients.

## Conclusion

This study is one of the first to assess the cognitive profile of NPC patients. Executive functions and attention were the most impaired functions while storage in the episodic memory was preserved in 61.5% of cases. Patients treated by miglustat remain globally stable for cognitive tests under treatment for several years. These results are interesting for a better understanding of cognitive impairments in NPC patients, for cognitive rehabilitation and for clinical follow-up and trial end points. Further prospective studies with complete neuropsychological examination are needed to confirm these data.

## Abbreviations

AD: Alzheimer’s disease
BNT: Boston naming test
CPP: Comité de protection des personnes (local ethic committee)
CRML: Centre de reference des maladies lysosomales (reference center for lysosomal diseases)
FAB: Frontal assessment battery
FCSRT: Free and cued selective reminding test
IC95: 95% Confidence interval
MMSE: Mini mental state evaluation
NPC: Niemann Pick type C
SPSS: Statistical package for social sciences software
